# Development of a questionnaire to assess the patient perspective regarding challenges in psoriatic arthritis treatment—a mixed-methods study

**DOI:** 10.1186/s42358-024-00414-7

**Published:** 2024-09-19

**Authors:** André Lucas Ribeiro, Júlia Andressa Tessari, Charles Lubianca Kohem, Penélope Esther Palominos, Rafael Mendonça da Silva Chakr

**Affiliations:** 1 Rheumatology Department, Hospital de Clínicas de Porto Alegre, Universidade Federal do Rio Grande do Sul, Ramiro Barcelos 2350, Porto Alegre, Rio Grande do Sul 90035-903 Brazil; 2 Universidade Federal do Rio Grande do Sul, Porto Alegre, Brazil; 3 Rheumatology Department, Hospital de Clínicas de Porto Alegre, Porto Alegre, Brazil; 4 Lifeplus Litoral Norte, Outliers Innovative Solutions, Xangrilá, Brazil

**Keywords:** Psoriatic arthritis, Healthcare disparities, Qualitative research, Socioeconomic factors, Health services accessibility

## Abstract

**Background:**

Limited data exist on psoriatic arthritis (PsA) treatment in lower-income regions, particularly from the patient perspective. This study explores the challenges faced by socioeconomically vulnerable PsA patients and the reasons for non-adherence to treatment guidelines. The main objective of the study is to develop a questionnaire to identify the primary challenges in PsA treatment adherence and to analyze its feasibility while simultaneously understanding the target population’s unique characteristics.

**Methods:**

We included PsA patients meeting the Classification Criteria for PsA (CASPAR), excluding those with other overlapping inflammatory diseases. The study, supported by two patient-research partners, began with focus groups to identify treatment challenges, leading to the creation of a 26-item questionnaire. Its reliability was verified using the test-retest method, targeting a percent agreement ≥ 0.8. Then, PsA patients at a rheumatology clinic completed the final survey.

**Results:**

The study involved 69 PsA patients. The final questionnaire contained 26-questions across five-domains, with a 92.2% agreement rate and an average completion time of 8.3 minutes. Diagnostic delays exceeded a year for 59% of patients and more than two years for 33%. Daily life disruptions affected 43.2% of patients, with 35.3% taking sick leave or retiring. Around 25% waited over 8 weeks for drug approval, and 17.6% required legal intervention to access medication. Drug dispensation issues impacted about 60% of patients. Furthermore, 66.7% lived far from their rheumatologist, with 49% traveling over an hour for appointments. Approximately 30% were unaware of the risks of methotrexatein relation to alcohol consumption and pregnancy.

**Conclusions:**

The questionnaire was feasible and reliable, with its results underscoring patient-centric challenges in PsA management, particularly concerning diagnostic delays and medication access, as well as daily life disruptions and misinformation. These findings emphasize the urgency for healthcare reforms aimed at improving diagnosis efficiency, patient education, and streamlined medication access, emphasizing the need for tailored initiatives to improve the healthcare experience for PsA patients.

**Supplementary Information:**

The online version contains supplementary material available at 10.1186/s42358-024-00414-7.

## Introduction

In Latin America, psoriasis prevalence ranges from 0.36 to 2.96%, with 19.5% of these patients developing psoriatic arthritis (PsA) [1, 2]. PsA is a heterogeneous disease that may affect multiple domains, thus requiring a multidisciplinary care team [3]. Despite its prevalence, it remains understudied in Latin America, with both a recent systematic literature review and the International League of Associations for Rheumatology (ILAR) underscoring the paucity of scientific literature in the region [4, 5].

There is a consensus that Latin American patients frequently face challenges in accessing adequate treatment due to educational and logistical barriers, such as a lack of rheumatologists and their unequal distribution, which results in significant delays in diagnosis and for beginning the treatment [6]. A 2023 systematic literature review highlighted issues hampering optimal care in Latin America, including opportunistic infections, diagnostic and treatment delay, difficulties related to the storage of biological medications, and the influence of socioeconomic factors on health outcomes. Yet, most studies overlooked the patient’s perspective, a critical component for fully understanding these issues [4].

In recent years, the emphasis on patient-reported outcomes (PROs) has increased, offering a better perspective into the multifaceted impacts of PsA [7]. Delayed treatment, for instance, compromises medication adherence and leads to increased radiographic progression and a reduced chance of achieving sustained, drug-free remission [8, 9]. While qualitative studies have grown in popularity to better understand these nuances, particularly after a consensus highlighted essential PsA domains for research [10], most do not address challenges related to healthcare access—a critical factor for successful treatment.

Given the pronounced challenges PsA patients encounter in accessing healthcare within Latin America, there is an urgent need to understand their distinct experiences and barriers. Since much of the existing research focuses primarily on clinical dimensions, it often overlooks the pivotal role that healthcare access imparts in a successful treatment. We aim to highlight the unique experiences of Brazilian PsA patients, examining barriers to optimal healthcare and adherence to guidelines. Through a patient-centric approach, our goal it both to close this knowledge gap and improve medical care, underscoring the irreplaceable role of patient narratives in creating a more responsive healthcare environment.

## Methods

The elaboration and application of the questionnaire followed the Standards for Reporting Qualitative Research (SRQR) [11]. Patients’ perspectives were prioritized throughout the development of the survey. Two patient-research partners (PRPs) assisted at all stages of the research to better understand and represent the patient viewpoint, aiming to create a patient-friendly questionnaire.

### Population

We enrolled adult patients with confirmed PsA from the rheumatology outpatient clinic of a single tertiary hospital between March 2023 and September 2023. Tertiary hospitals in Brazil predominantly manage and treat advanced cases, establishing themselves as referral centers for complex medical conditions. Eligibility criteria included the need to meet ClASsification criteria for Psoriatic ARthritis (CASPAR) [12], the capability to complete a questionnaire, and a signed informed consent form. Importantly, illiteracy was not a barrier to inclusion; patients who could not read were still eligible if they had a family member available to assist in filling out the questionnaire. Exclusion criteria were limited to patients aged less than 18 years and to patients with concurrent inflammatory diseases (e.g., systemic lupus erythematous, Crohn’s’ disease, etc.), as these could potentially influence patients’ experiences.

In brief, we anticipated a need for 70 participants across all three phases of the study. The population size for focus groups was determined by the saturation technique, forming new groups until no new themes emerged in two consecutive sessions [13]. For the test-retest phase, a standard ratio of one patient per five questionnaire items was used [14]. The final phase involved convenience sampling, including all eligible PsA patients attending the clinic over three months. Participants were unique to each phase to prevent response overlap.

To better characterize our cohort, we utilized a two-phase data collection method. Initially, during the focus groups and test-retest stages, clinical and demographic details were gathered. This involved reviewing electronic medical records (EMRs) and administering a sociodemographic questionnaire. Additionally, we screened for fibromyalgia using the Fibromyalgia Rapid Screening Tool (FiRST) and for depression with the Patient Health Questionnaire 2 (PHQ-2) [15, 16]. Fibromyalgia was specifically assessed due to its known impact on disease activity, quality of life, and difficulty in facing daily challenges, which could influence patients’ answers [17, 18]. For the final phase of data collection, we exclusively relied on EMRs, omitting the supplementary questionnaires and screening tools for efficiency and feasibility.

### Focus groups

The initial phase of the project involved the development of focus groups, a strategy frequently employed in research for hypothesis generation [19]. The principle of saturation dictated the number of sessions, meaning that we conducted interviews with three to four PsA patients until no new information emerged in two consecutive sessions [13]. The objective was to identify the most common difficulties faced by patients while seeking health care specifically for PsA. An interview script, co-developed with PRPs, guided all sessions. Initially, participants reflected on challenges faced in obtaining PsA treatment, from pre-diagnosis to present. Specific topics (like treatment and referral times) were prompted if omitted. Near the session’s end, another open question asked about any unmentioned treatment-related issues.

Every session was audio-recorded and subsequently transcribed into a systematically coded Word document. This transcription was then translated into an Excel spreadsheet and thematically examined by two core team members, a physician and a patient. This duo engaged in a collaborative analysis to identify particular areas of treatment concerns voiced by patients. In cases of disagreements that could not be solved by consensus, a third team member provided a resolution.

### Development of the questionnaire

The questionnaire was developed based on the domains identified in the focus groups. The questions were presented in a logical sequence, with general queries preceding the more specific ones [20]. The PRPs were instrumental in this process, participating both in the initial formulation of the survey and in the cognitive interviewing to ensure that the respondents would comprehend the questions as the designers intended them to be [21]. The questions focused on the past 2 years to decrease memory bias and to capture the current panorama of the Brazilian healthcare system.

After the cognitive interviews, we presented the questionnaire to an 11-year-old child to assess for clarity and readability, adhering to the guidelines set forth by the American Medical Association and the National Institute of Health. These organizations recommend that patient materials target a reading level of 6th to 8th grade, respectively, underscoring the importance of clear health communication [22].

### Test-retest

The test-retest method evaluates reliability by having a select group of patients complete the survey on two occasions [14]. In keeping with standard practices, we used a ratio of one patient for every five questionnaire items, with a 10 to 14-day interval between each survey completion [14]. We then calculated the percent agreement, which represents the proportion of consistent answers, to ascertain stability and consistency over time. A percent agreement score exceeding 0.8 was deemed indicative of strong test-retest reliability. During this phase, we also recorded the time taken to complete the questionnaires and gathered feedback on their understandability through an open-ended question.

### Data collection

During the final phase, we utilized convenience sampling to enlist eligible PsA patients visiting the outpatient clinic. Over a period of three months, approximately 4–5 patients per week (totaling 12 weeks) consecutively completed the questionnaire, aiming to gather data to characterize our cohort. Patients independently filled out the questionnaires, ensuring minimal bias and no direct involvement from the research team.

This convenience sampling approach was chosen to balance the qualitative principles of in-depth exploration with the feasibility constraints of conducting research at a single center. By engaging all available patients during this period, we aimed to maximize the response rate and ensure that the questionnaire captured a broad spectrum of patient experiences. The methodology employed reflects qualitative research principles, focusing primarily on the depth and richness of the data collected, rather than on the statistical power calculations typically used in quantitative studies. Further validation of the questionnaire across multiple centers in Brazil is anticipated to enhance the generalizability of our findings and confirm the consistency of our results across varied demographic and socioeconomic contexts.

The analysis focused on descriptive statistics, including frequency of occurrence and mean values, to effectively characterize the patient sample.

### Ethical considerations

The project was approved by the local ethics committee (Ethical Evaluation Presentation Certificate—CAAE—number 66326122.6.0000.5327) and was developed in accordance with the General Data Protection Law Compliance Statement (LGPD). All patients from the focus groups and from the test-retest phases willingly signed the informed consent form. For the data collection phase, however, the informed consent form requirement was waived by the ethics committee due to the absence of demographic data collection.

## Results

### Population characteristics

We included a total of 69 PsA patients: 12 in the focus group, 6 in the test-retest phase, and 51 in the data collection. Table 1 outlines the demographic and clinical characteristics of the 18 patients included in the focus group and test-retest phases. Our cohort was composed of 55.5% females (*n* = 10), predominantly White (88.9%, *n* = 16), with an average age of 58.5 years (SD = 12.0). Most were retired (61.1%, *n* = 11) and only 11.1% had attended college. The majority of patients (94.5%) had a household income below R$ 5000.00, placing them in the lower income brackets in Brazil (C and DE) [23], thereby making them socioeconomically vulnerable and primarily dependent on the universal healthcare system (SUS—Sistema Único de Saúde) for healthcare. We observed an average diagnosis delay of 2.7 years for psoriasis and of 5.4 years for PsA. Patients with fibromyalgia showed higher depression rates (71.4% vs. 27.2%), more pain (7.7 vs. 5.2 out of 10), higher mean DAPSA (18.6 vs. 10.7), and lower proportion of patients achieving minimal disease activity (MDA—0% vs. 30%). Regarding treatment, 73% were using conventional synthetic disease modifying anti-rheumatic drugs (csDMARDs) and 55% were on biologic therapy (bDMARDs). This sample reflects the characteristics of our larger survey cohort (*n* = 51), detailed in Supplementary Table S1.

**Table 1:** Demographic and clinical data of focus group and test-retest patients

Sample characteristics	
Sex, percentage (*n*)	
Male	44.5% (*n* = 8)
Female	55.5% (*n* = 10)
Race and ethnicity, percentage (*n*)	
White	88.9% (*n* = 16)
Black	11.1% (*n* = 2)
Age, mean (SD)	58.5 years (SD 12.0)
Marital status, percentage (*n*)	
Single	27.7% (*n* = 5)
Married	55.5% (*n* = 10)
Divorced	16.7% (*n* = 3)
Education, percentage (*n*)	
Incomplete elementary school	33.3% (*n* = 6)
Complete elementary school	16.7% (*n* = 3)
Complete high-school	38.9% (*n* = 7)
Complete college	11.1% (*n* = 2)
Occupation, percentage (*n*)	
Full-time job	16.7% (*n* = 3)
Partial-time job	11.1% (*n* = 2)
Unemployed	11.1% (*n* = 2)
Retired^a^	61.1% (*n* = 11)
Family income, percentage (*n*)	
<2000	16.7% (*n* = 3)
2000–3000	33.3% (*n* = 6)
3000–5000	44.4% (*n* = 8)
5000–10,000	5.5% (*n* = 1)
Age at psoriasis diagnosis, mean (SD)	40.2 years (14.2)
Age at PsA diagnosis, mean (SD)	46.2 years (13.1)
Psoriasis duration before diagnosis, mean (SD)	2.7 years (3.7)
PsA duration before diagnosis, mean (SD)	5.4 years (4.5)
CASPAR score, mean (SD)	4.8 (0.7)
LEI, mean (SD)	0.8 (1.1)
DAPSA, mean (SD)	14.5 (10.0)
MDA, percentage (*n*)	
Yes	22.2% (*n* = 4)
No	77.8% (*n* = 14)
Modified HAQ, mean (SD)	1.0 (0.8)
Pain NRS, mean (SD)	6.2 (2.7)
Global disease NRS, mean (SD)	6.5 (2.8)
Fatigue NRS, mean (SD)	5.5 (3.6)
Stiffness NRS, mean (SD)	3.2 (2.5)
Skin NRS, mean (SD)	5.4 (3.4)
Active psoriasis, percentage (*n*)	
Yes	83.3% (*n* = 15)
No	16.6% (*n* = 3)
History of dactylitis, percentage (*n*)	
Yes	44.4% (*n* = 8)
No	55.5% (*n* = 10)
Rheumatoid factor, percentage (*n*)	
Yes	16.6% (*n* = 3)
No	83.3% (*n* = 15)
Radiographic enthesophytes, percentage (*n*)	
Yes	77.8% (*n* = 14)
No	22.2% (*n* = 4)
Depression, percentage (*n*)	
Yes	38.9% (*n* = 7)
No	61.1% (*n* = 11)
Fibromyalgia, percentage (*n*)	
Yes	44.4% (*n* = 8)
No	55.5% (*n* = 10)
LTBI, percentage (*n*)^b^	
Yes	40% (*n* = 4)
No	60% (*n* = 6)
csDMARDs, percentage (*n*)	73.2% (*n* = 13)
Methotrexate	66.7% (*n* = 12)
Leflunomide	5.5% (*n* = 1)
bDMARDs, percentage (*n*)	
Yes	55.5% (*n* = 10–7 TNFi and 3 IL-17i)
No	44.4% (*n* = 8)

*N* number, *SD* standard deviation, *CASPAR* ClASsification criteria for Psoriatic Arthritis, *LEI* Leeds enthesitis index, *DAPSA* Disease Activity in PSoriatic Arthritis, *MDA* minimal disease activity, *HAQ* health assessment questionnaire, *NRS* numerical rating scale, *LTBI* latent tuberculosis infection, *csDMARDs* conventional synthetic disease modifying anti-rheumatic drugs, *bDMARDs* biologic disease modifying anti-rheumatic drugs, *TNFi* tumor necrosis factor inhibitors, *IL-17i* interleukin 17 inhibitors

^a^3 patients had early retirement due to medical causes

^b^Only patients using biologic are routinely tested

### Focal group and test-retest phases

We conducted 4 focus groups comprising three to four patients each, totaling 12 patients. During these discussions, five key themes emerged: difficulties in disease diagnosis, daily life disruptions secondary to PsA, drug dispensation issues, healthcare access obstacles, and misinformation. Based on these insights, a questionnaire with 26 questions covering these domains was developed. We provide the original Portuguese questionnaire and its English translation, along with response proportions, in Supplementary Tables 2 and 3. The questionnaire’s average completion time was 8.3 min (SD = 3.1 min), with a retest interval averaging 10.4 days (SD = 2.0 days), and a 92.2% agreement rate.

Patients shared compelling accounts of their PsA journey, illustrating the myriad challenges they faced from diagnosis to treatment. Their stories highlighted the often ambiguous nature of early PsA symptoms and the consequent diagnostic hurdles. One notable example was Patient A, a 49-year-old male, who detailed his frustrating experience with persistent pain in his hands and elbows and uncertainty about its cause: “There was a lot of pain in my hands, in my elbows, so I did blood work and X-rays, did everything, and I still didn’t know what it was, nothing showed up.” This narrative underscores the complexity and difficulties of early PsA diagnosis when radiographs are still normal.

Patients frequently mentioned how PsA affected their ability to perform everyday tasks and work duties. Patient E, a 33-year-old male, shared: “I still have difficulties in picking up a glass from time to time because the hand doesn’t bend well… I worked as a driver and my hands would become all swollen. It was very difficult and I had to change my job.” These accounts vividly depict the disease’s impact on daily living and employment.

The challenges in obtaining medication were another significant concern. Patient B, a 66-year-old woman, stated: “The medication delay through the State Pharmacy is constant because the delivery there is just not right. There are always problems with the delivery of medication… It’s never regular.” Similarly, Patient A mentioned, “I’ve already gone 4 months without injections. Four months and nothing came.” These experiences highlight systemic issues in medication supply and access.

Transportation to appointments and geographical barriers emerged as prominent issues. Patient D, a 61-year-old male, noted: “It takes me 3 hours to get to the appointment.” Additionally, Patient E, a 33-year-old man, remarked on the logistical challenges: “But I live in Livramento, 9 hours by bus, and I had to come here just to make an appointment. See if that makes sense, it doesn’t.” These statements emphasize the difficulties in accessing routine care for many patients.

Adverse effects of medications were also a common concern. Patient C, a 66-year-old woman, described her experience: “Then it was horrible, those eight pills for a day (methotrexate), it was a lot of nausea and a lot of vomiting for two days, horrible. It made me very sick; I vomited a lot when it was started.” This highlights the need for careful consideration of treatment side effects in managing PsA.

### Questions regarding diagnostic issues

**Fig. 1:**
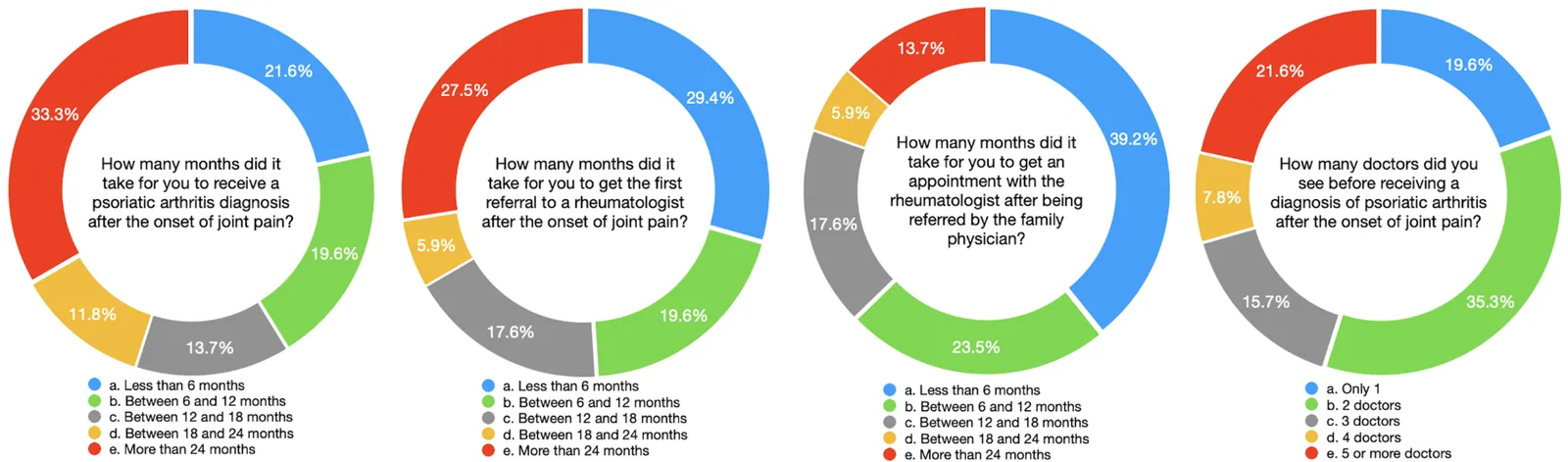
Questions regarding diagnostic issues

There was a significant delay in both diagnosis and specialist referrals (summarized in Fig. 1). Approximately 59% of respondents waited over a year after the onset of joint pain before receiving a PsA diagnosis, with 33% waiting over 2 years. A minority (21.6%) were diagnosed within the first six months of symptoms. Regarding referral times, 27.5% had to wait over two years to be referred to a rheumatologist after initially reporting joint pain. After the referral, 36% waited more than 1 year for an appointment. The path to a correct diagnosis often required consulting multiple physicians: 80.4% required seeing more than one physician before arriving at a PsA diagnosis.

### Questions related to difficulties in daily life

**Fig. 2:**
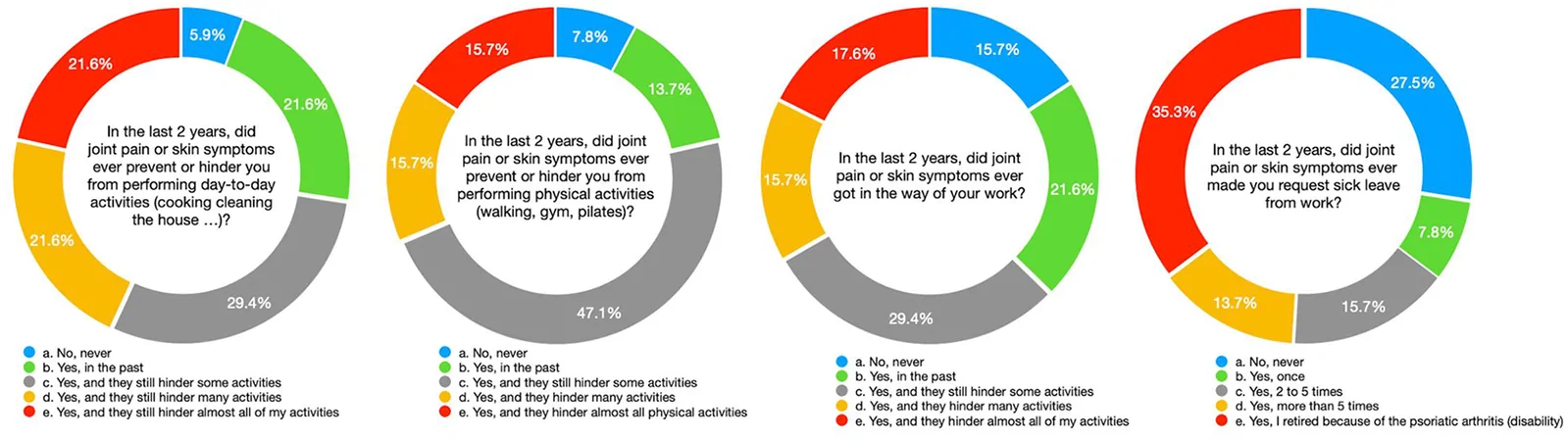
Questions related to difficulties in daily life

A considerable portion of respondents indicated significant daily life disruptions caused by PsA (Fig. 2). Around 30% of the respondents mentioned that the symptoms still obstructed some activities (e.g., cooking, cleaning), while 43.2% felt many or almost all such activities were negatively affected. The impact on physical activities was also pronounced, with 31.4% affirming that many or almost all physical activities were limited due to the disease. With regard to work, 29.4% mentioned that the symptoms still affected some of their duties, with 33.3% indicating that many or almost all tasks were impacted. Finally, 35.3% reported taking sick leave or early retirement because of the disease, underlining its impact on the professional life.

### Questions regarding drug dispensation challenges

**Fig. 3:**
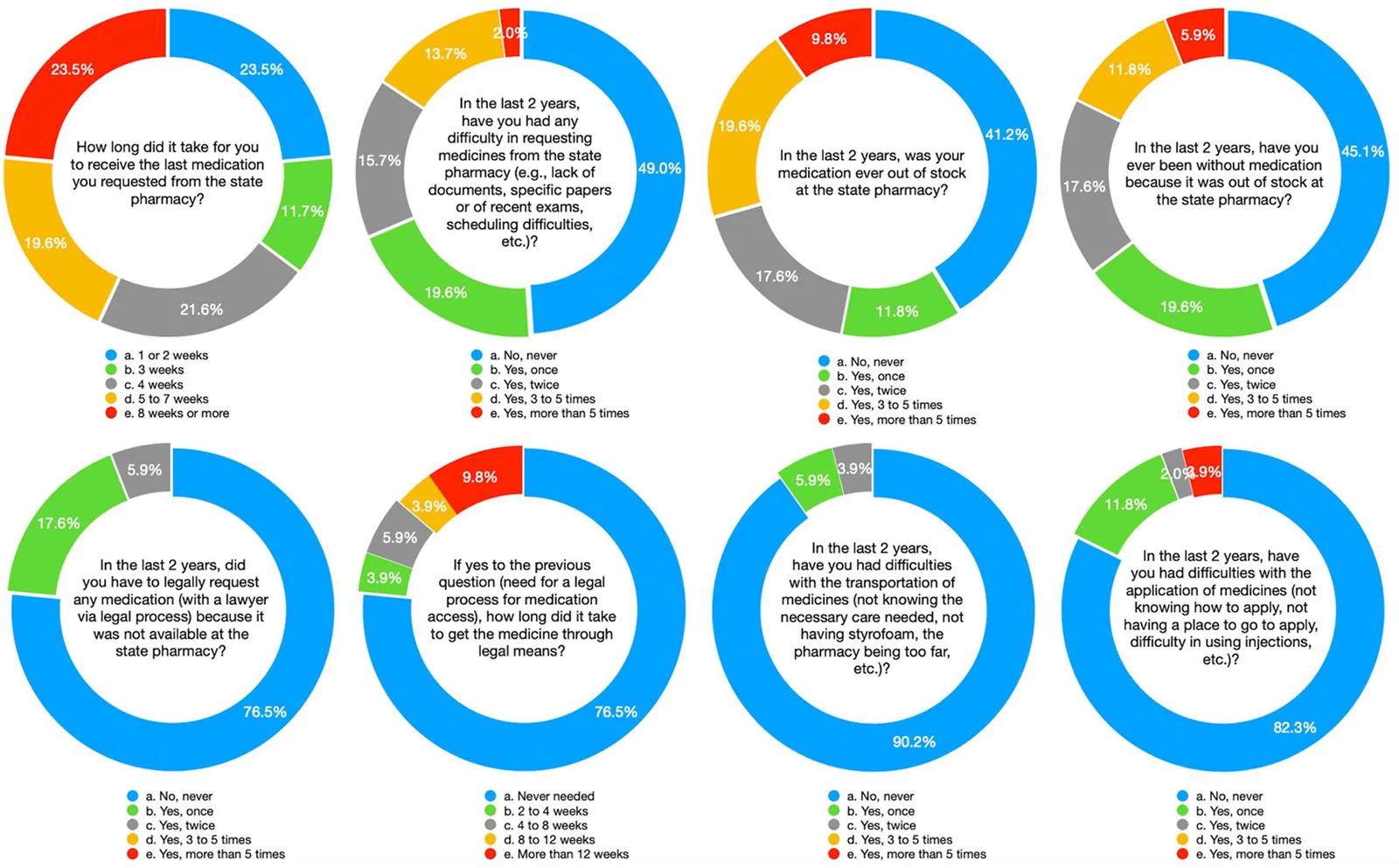
Questions related to drug dispensation issues

Waiting times to start treatment after drug requisition were 5 to 7 weeks for 19.6% of respondents and of 8 or more weeks for 23.5% of them (Fig. 3). About half (49%) stated they had never faced difficulties when requesting medicines; however, 15.7% faced these issues twice, and 13.7% experienced them between 3 and 5 times. Stock-related concerns were apparent: 19.6% faced medication shortages 3 to 5 times, and 9.8% experienced it more than 5 times in the past 2 years. About 17.6% had to legally request medications (i.e., judicialization) at least once due to medication unavailability through the universal healthcare. The judicialization process usually took longer, with 9.8% of the respondents waiting more than 12 weeks to receive the drugs through legal channels. Logistical challenges were less frequent: 90.2% faced no difficulties with medicine transportation, and 82.3% had no issues with medication application. However, 11.8% faced difficulties with medication self-application.

### Questions related to issues with routine medical care

**Fig. 4:**
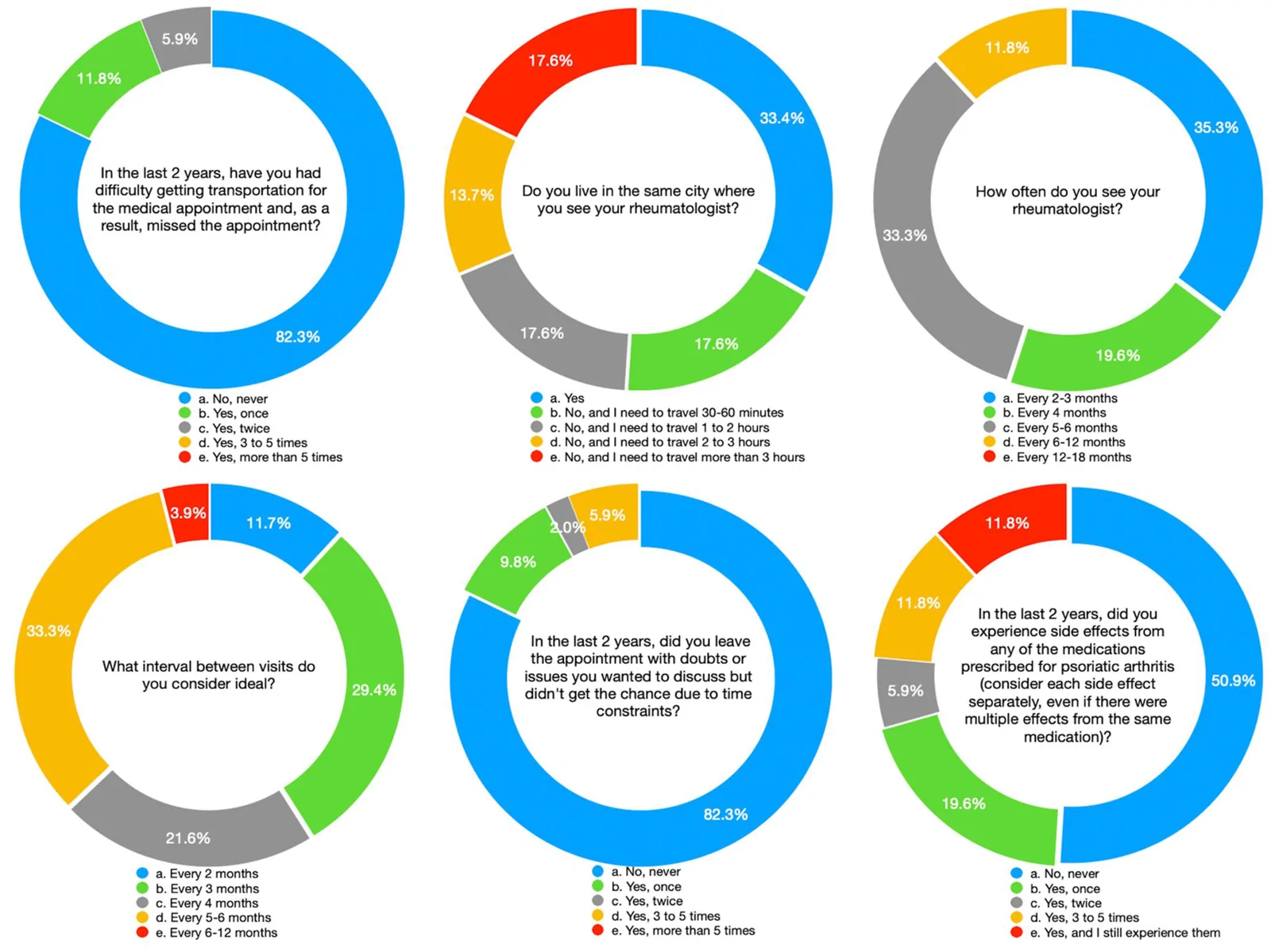
Questions related to routine medical care

When focusing on routine medical care for patients with PsA, transportation and geographical challenges emerge as prominent issues, as shown in Fig. 4. The majority of patients (66.7%) do not live in the same city as their rheumatologist, with 49% of them taking at least 1 h to go to their appointment. About 11.8% of the patients had missed an appointment due to transportation problems. The frequency of the appointments ranged from every 2–3 months (35.3%) to every 5–6 months (33.3%) and 6–12 months (11.8%). The patients’ preferred interval was every 2–3 months in 41.1% of cases. Most patients (82.3%) felt adequately informed post-appointment. Recurrent medication side effects were reported by 49% of respondents.

### Question related to lack of information/misinformation

**Fig. 5:**
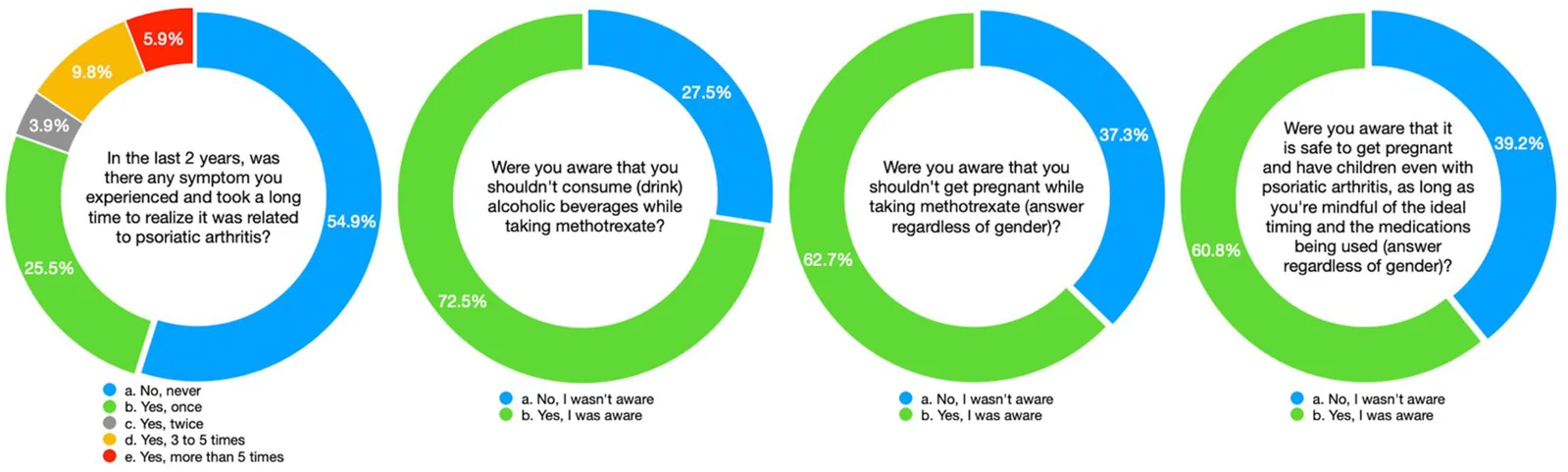
Questions related to misinformation

Over half (54.9%) of respondents correctly identified all symptoms related to the condition, but many experienced delays in attributing certain symptoms to it. While most patients were aware of the risks associated with consuming alcohol (72.5%) or getting pregnant (62.7%) while on methotrexate, 30–40% were still unaware of these risks. Notably, the prevalence of methotrexate use was 49%. Furthermore, 39.2% of respondents were concerned that PsA posed a risk to safely conceiving children, leading to concerns about potential reproductive complications (Fig. 5).

## Discussion

In light of the considerable challenges in managing PsA across Latin America, our research provides pivotal insights into the unique experiences and obstacles faced by PsA patients in Brazil. To the best of our knowledge, this study represents the first of its kind in Brazil to provide such a comprehensive exploration of the challenges faced by PsA patients. By emphasizing patient perspectives, an aspect often neglected in existing research, our study enriches the understanding of PsA’s diverse impacts. This focus aligns with the increasing recognition of the value of PROs, and it directly addresses the critical need for accessible healthcare, a key determinant in effective PsA management. Our findings underscore the necessity of incorporating patient experiences into medical care, thereby contributing significantly to the development of a healthcare system in Latin America that is more responsive to patient needs.

In the development of our questionnaire, focus groups were instrumental in identifying key domains of patient experience that were then transformed into specific survey questions. This approach guaranteed that the questionnaire was firmly grounded in actual patient concerns. The involvement of PRPs in developing the focus group script, helping with formulating the questions, and ensuring their clarity and patient-friendliness through cognitive interviews, further enhanced our survey. Moreover, the questionnaire was specifically designed for the Brazilian healthcare context, rather than being an adaptation of foreign models, which significantly increased its relevance to the local cohort.

Our survey adhered to the SRQR guidelines, in accordance with the Enhancing the QUAlity and Transparency Of health Research (EQUATOR) Network recommendations for qualitative research [24]. Additionally, the Outcome Measures in Rheumatology (OMERACT) standards were also considered in its development [25]. The survey was considered feasible, as it was easily understood and required a short completion time. In terms of face validity, it had input from PsA specialists and patients in Brazil, ensuring that it was highly relevant and appropriate for the local cohort. Cognitive interviewing with PRPs refined these inputs, which helped ensure that the questions were interpreted as intended and resonated with the experiences of PsA patients. Construct validity was achieved by incorporating diverse PsA-related domains based on the existing literature in the field. The active involvement of PRPs and PsA experts in the development process further authenticated the relevance and representativeness of the questionnaire, improving its ability to capture the multifaceted experience of living with PsA in Brazil.

The clinical data from our cohort distinctly highlight the prognostic impact of fibromyalgia on disease outcomes, with a marked correlation between its presence and reduced remission rates, consistent with prior studies assessing fibromyalgia’s burden in PsA [26, 27]. These findings emphasize the importance of employing fibromyalgia screening tools, which are available in Brazilian Portuguese [28]. The clear link between fibromyalgia and detrimental patient outcomes underscores the necessity for a multidisciplinary care team (MDC) approach, ensuring an individualized treatment strategy [29]. Given that median healthcare costs for patients with fibromyalgia can be up to five times higher, refining its management could reduce the system’s economic strain significantly [30]. Finally, while the high prevalence of fibromyalgia may have biased our questionnaire, the structured and categorical nature of our questions aimed to minimize its impact on assessing patient experiences.

A significant diagnostic delay was observed in our study. Patients faced a waiting period of 2.7 years for a psoriasis diagnosis and an extended time of 5.4 years for PsA. Our survey further revealed that 59% of respondents waited over a year after the onset of joint pain to receive a PsA diagnosis. These results emphasize the need for the implementation of streamlined referral protocols to decrease waiting times, as emphasized by recent guidelines [31]. The implementation of well-defined referral protocols and telehealth consultations with specialists could simultaneously mitigate diagnostic delays and reduce healthcare costs [32, 33]. Additionally, increasing public knowledge about PsA, implementing screening tools in psoriasis clinics, and devising educational initiatives for primary care doctors have been shown to improve the accuracy and speed of disease diagnosis [34–36]. Finally, expansion of primary healthcare, including the incorporation of lay community health agents and interdisciplinary care teams, has been shown to improve mortality and decrease healthcare inequalities in Brazil [37, 38].

Concerns also arise from drug availability and inconsistent dispensation. Our data indicate prolonged wait times for medication approval. However, albeit prolonged, these delays fare better than the 75-day average time reported in other studies carried out in Brazil, underscoring disparities among regions [39]. In our survey, 17.6% of the patients were forced to resort to judicialization to obtain medications due to their unavailability in the SUS, which is a time-consuming and costly method that has proliferated throughout Latin America [40]. Addressing these challenges involves streamlining the process of incorporating new medications into the Clinical Protocols and Therapeutic Guidelines (PCDT), which is crucial for ensuring their availability in the SUS. For example, from 2017 to 2020, the average time for a medication to be incorporated into the PCDT after submission to Conitec (Comissão Nacional de Incorporação de Tecnologias) was 217.6 days [41]. Following this approval, there was an additional delay of about 372.9 days before these medications became accessible to patients [41]. Reducing these protracted timelines is critical to improving medication access, decreasing healthcare costs linked to judicialization, and enhancing the efficiency of the SUS.

After drug approval, roughly 50% of the patients reported experiencing irregular drug dispensation, which is linked to adverse events, increased mortality rates, and rising costs [42]. However, institutions with dedicated assisted therapy centers offer a promising solution in Latin America. By centralizing drug distribution for all enrolled patients, this model ensures consistent drug supply. This streamlined approach, adopted by several tertiary centers, has demonstrated the dual benefits of regular drug supply and cost reductions through the efficient use of medications [43]. Ultimately, central oversight of medication dispensation could enhance the current decentralized approach in Brazil, as national supply redistributions have shown potential in addressing shortages [44].

The majority of patients reported no issues with drug transportation and storage, a positive shift from past studies [45, 46]. However, about 18% struggled with drug application, often due to challenges with self-administration, emphasizing the need for more user-friendly medication designs. In rheumatology, where patients often have physical limitations, easier application methods are crucial. For instance, while methotrexate in Brazil is provided in a 2 ml bottle, a prefilled, auto-injectable pen, which is available in several countries, has been shown to improve the user experience [47]. Additionally, implementing measures to reduce pain from subcutaneous injections could also enhance medication adherence [48].

Given the routine medical care required for patients with PsA, it is alarming that 66.7% of patients reside in a different city than their rheumatologists, and that 18% reported missing an appointment in the last two years due to lack of available transportation. This underscores the pressing need to better distribute rheumatologists across regions. The current prevalence of rheumatologists in Latin America stands at 1 per 106,838 inhabitants, which is considerably lower than the recommended minimum of 1 rheumatologist per 50,000 inhabitants [49, 50]. This disparity is amplified by the urban-rural divide in specialist availability [51]. To increase the availability of rheumatologists in smaller centers and rural areas, solutions might involve offering financial incentives, improving living conditions and local medical facilities, and giving preference to rural students in health programs. Implementing these strategies has previously led to improved practitioner retention in underserved regions [52–54].

Finally, our survey underscores a significant knowledge gap in the patients’ understanding of their condition. While a majority could identify PsA symptoms, many failed to recognize specific symptoms. Moreover, there is a significant lack of awareness regarding the impact of certain behaviors, such as the risks associated with alcohol consumption, including liver injury from alcohol and methotrexate interaction, and the teratogenic risks during pregnancy while on methotrexate. This is particularly alarming given the widespread use of methotrexate (49%) among our respondents. The EULAR underscores the significance of patient education and has issued recommendations to improve it [55]. Physicians can address this gap through clear communication, avoiding medical jargon, and by encouraging patient queries [56]. In doing so, healthcare providers can mitigate the risks associated with limited patient health literacy while simultaneously strengthening the patient-provider relationship and improving outcomes.

While this study offers valuable insights into the patient perspective on PsA treatment challenges in Brazil, it is important to acknowledge its limitations. Conducted at a single tertiary care center in the South of Brazil, the research setting allowed for controlled and detailed data collection and analysis but may not fully capture the broader demographic and socio-economic spectrum of PsA patients across the nation. Given the diversity of Brazil’s population and healthcare infrastructure, the experiences and healthcare access challenges faced by patients in different regions may vary significantly. Furthermore, the relatively small sample size, while adequate for initial explorations within our research setting, limits the generalizability of our findings and calls for the risk of selection bias. Thus, these results should be seen as preliminary and interpreted with caution. Additionally, the study’s reliance on patient-reported data introduces the potential for recall bias, particularly as participants were asked to reflect on their experiences over the past two years, which might affect the accuracy of data on symptom onset and diagnosis and treatment timelines. Finally, the exclusion of patients with other concurrent inflammatory diseases, although intended to reduce confounding, might have limited our understanding of the full spectrum of challenges faced by PsA patients with comorbid conditions. These limitations highlight the need for further validation of our findings across multiple centers in Brazil to enhance the robustness and applicability of the results and ensure that the developed questionnaire and ensuing conclusions are relevant and adaptable to various Brazilian contexts.

In conclusion, our study highlights the challenges faced by patients, spanning from delayed diagnosis to daily functional limitations and systemic barriers in medication access. These findings emphasize the disease’s multifaceted impact, extending beyond physical symptoms to include work-related issue and logistical challenges. It is paramount that new healthcare policies actively pursue initiatives focused on patient education, early diagnosis, and streamlined treatment access. Implementing a patient-centric strategy can translate these insights into practical improvements, thereby enhancing patient care and overall quality of life.

## Electronic supplementary material

Below is the link to the electronic supplementary material.

Supplementary Material 1

## Data Availability

All data generated or analyzed during this study are included within the published article and its additional files in the supplementary material.
