# Supplementary material for: Development of a questionnaire to assess the patient perspective regarding challenges in psoriatic arthritis treatment—a mixed-methods study
Source: Adv Rheumatol. 2024 Sep 19;64:72. doi: 10.1186/s42358-024-00414-7 (PMC13488806; doi:10.1186/s42358-024-00414-7)
Supplement: Supplementary file 1 [file 42358_2024_414_MOESM1_ESM.docx]

**Supplementary Material**

**Index**

[Table 1S. Demographic and clinical data of patients that responded the questionnaire.	3](#_Toc154662997)

[Table 2S. Original Questionnaire in Portuguese.	5](#_Toc154662998)

[Table 3S. Translated Questionnaire in English with Percentage of Answers per Question.	9](#_Toc154662999)

# Table 1S. Demographic and clinical data of patients that responded the questionnaire.

| Sample characteristics |  |
| --- | --- |
| Sex, percentage (n)  Male  Female | 29.4% (n = 15)  70.6% (n = 36) |
| Race and ethnicity, percentage (n)  White  Black | 92.2% (n = 47)  7.8% (n = 4) |
| Age, mean (SD) | 58.1 years (SD 11.5) |
| Marital status, percentage (n)  Single  Married  Divorced  Widowed | 23.5% (n = 12)  64.7% (n = 33)  7.8% (n = 4)  3.9% (n = 2) |
| Education, percentage (n)  Incomplete elementary school  Complete elementary school  Complete high-school  Complete college | 33.3% (n = 17)  23.5% (n = 12)  37.2% (n = 19)  5.9% (n = 3) |
| Active psoriasis, percentage (n)  Yes  No | 43.1% (n = 22)  56.9% (n = 29) |
| History of dactylitis, percentage (n)  Yes  No | 37.3% (n = 19)  62.7% (n = 32) |
| Radiographic enthesophytes, percentage (n)  Yes  No | 60.8% (n = 31)  39.2% (n = 20) |
| Depression, percentage (n)  Yes  No | 51% (n = 26)  49% (n = 25) |
| Fibromyalgia, percentage (n)  Yes  No | 27.5% (n = 14)  72.5% (n = 37) |
| LTBI, percentage (n)^b^  Yes  No | 48.6% (n = 18)  51.4% (n = 19) |
| csDMARDs, percentage (n)  Methotrexate  Leflunomide  Sulfasalazine | 60.8% (n = 31)  49.0% (n = 25)  9.8% (n = 5)  2% (n = 1) |
| bDMARDs, percentage (n)  Yes  No | 60.8% (n = 31 – 13 TNFi / 12 IL-17i / 2 iJAK / 3 IL12/23)  39.2% (n = 20) |

^a^Only patients using biologic, or that used biologics in the past, are routinely tested

N, number; using biologic, or that used biologics in the past, are routinely tested

N, number; SD, standard deviation; LTBI, latent tuberculosis infection; csDMARDs, conventional synthetic disease modifying anti-rheumatic drugs; bDMARDs, biologic disease modifying anti-rheumatic drugs; TNFi, tumor necrosis factor inhibitors; IL-17i, interleukin 17 inhibitors; iJAK, JAK inhibitor; IL12/23, interleukin 12/23.

# **Table 2S.** Original Questionnaire in Portuguese.

**Questões relacionadas ao diagnóstico:**

1. Quantos meses demorou para você receber o diagnóstico de artrite psoriásica após o início das dores articulares (nas juntas)?
   a. Menos de 6 meses
   b. Entre 6 e 12 meses
   c. Entre 12 e 18 meses
   d. Entre 18 e 24 meses
   e. Mais de 24 meses
2. Quantos meses demorou para você receber o primeiro encaminhamento para um reumatologista após o início das dores articulares (nas juntas)?
   a. Menos de 6 meses
   b. Entre 6 e 12 meses
   c. Entre 12 e 18 meses
   d. Entre 18 e 24 meses
   e. Mais de 24 meses
3. Quantos meses demorou para você conseguir a consulta com o reumatologista após o encaminhamento médico pelo posto de saúde?
   a. Menos de 6 meses
   b. Entre 6 e 12 meses
   c. Entre 12 e 18 meses
   d. Entre 18 e 24 meses
   e. Mais de 24 meses
4. Por quantos médicos você passou até receber o diagnóstico de artrite psoriásica após o início das dores articulares (nas juntas)?
   a. Apenas 1
   b. 2 médicos
   c. 3 médicos
   d. 4 médicos
   e. Mais do que 5 médicos

**Questões de dificuldades na rotina:**

5. Nos últimos 2 anos, os sintomas articulares (dor nas juntas) ou cutâneos (na pele) alguma vez o impediram, ou dificultaram, a realização de atividades do dia-a-dia (cozinhar, limpar a casa...)?
   a. Não, nunca
   b. Sim, no passado
   c. Sim, e ainda dificultam a realização de algumas atividades
   d. Sim, e ainda dificultam a realização de muitas atividades
   e. Sim, e ainda dificultam a realização de quase todas as minhas atividades
6. Nos últimos 2 anos, os sintomas articulares (dor nas juntas) ou cutâneos (na pele) alguma vez o impediram ou dificultaram a realização de atividades físicas (caminhar, realizar academia, pilates)?
   a. Não, nunca
   b. Sim, no passado
   c. Sim, e ainda dificultam a realização de algumas atividades
   d. Sim, e ainda dificultam a realização de muitas atividades
   e. Sim, e ainda dificultam a realização de quase todas as atividades físicas
7. Nos últimos 2 anos, os sintomas articulares (dor nas juntas) ou cutâneos (na pele) alguma vez o impediram ou dificultaram o seu trabalho?
   a. Não, nunca
   b. Sim, no passado
   c. Sim, e ainda dificultam a realização de algumas atividades
   d. Sim, e ainda dificultam a realização de muitas atividades
   e. Sim, e ainda dificultam a realização de quase todas minhas atividades
8. Nos últimos 2 anos, os sintomas articulares (dor nas juntas) ou cutâneos (na pele) alguma vez fizeram você solicitar afastamento do trabalho?
   a. Não, nunca
   b. Sim, 1 vez
   c. Sim, de 2 a 5 vezes
   d. Sim, mais de 5 vezes
   e. Sim, me encostei (ou aposentei) por causa da artrite psoriásica

**Questões de dispensação de remédios:**

9. Quanto tempo demorou para você receber o último remédio que encaminhou pela farmácia do estado?
   a. 1 ou 2 semanas
   b. 3 semanas
   c. 4 semanas
   d. 5 a 7 semanas
   e. 8 semanas ou mais
10. Nos últimos 2 anos, você teve alguma dificuldade em encaminhar e/ou solicitar os remédios pela farmácia do estado (falta de documentos, falta de papeis específicos, falta de exame recente, dificuldade em agendamentos, ou outras)?
    a. Não, nunca
    b. Sim, 1 vez
    c. Sim, 2 vezes
    d. Sim, de 3 a 5 vezes
    e. Sim, mais de 5 vezes
11. Nos últimos 2 anos, alguma vez o seu remédio ficou em falta na farmácia do estado?
    a. Não, nunca
    b. Sim, 1 vez
    c. Sim, 2 vezes
    d. Sim, de 3 a 5 vezes
    e. Sim, mais de 5 vezes
12. Nos últimos 2 anos, você ficou alguma vez sem o remédio por ele estar em falta na farmácia do estado?
    a. Não, nunca
    b. Sim, 1 vez
    c. Sim, 2 vezes
    d. Sim, de 3 a 5 vezes
    e. Sim, mais de 5 vezes
13. Nos últimos 2 anos, você teve que encaminhar algum remédio pela via judicial (com advogado) por ele não estar disponível na farmácia do estado?
    a. Não, nunca
    b. Sim, 1 vez
    c. Sim, 2 vezes
    d. Sim, de 3 a 5 vezes
    e. Sim, mais de 5 vezes
14. Se sim à pergunta anterior (pergunta 13), quando tempo demorou para conseguir o remédio pela via judicial?
    a. Até 2 semanas
    b. 2 semanas a 4 semanas
    c. 4 semanas a 8 semanas
    d. 8 semanas a 12 semanas
    e. Mais do que 12 semanas
15. Nos últimos 2 anos, você teve dificuldades com o transporte dos remédios (não saber quais os cuidados, não ter isopor, o local ser muito longe, ou outras)?
    a. Não, nunca
    b. Sim, 1 vez
    c. Sim, 2 vezes
    d. Sim, de 3 a 5 vezes
    e. Sim, mais de 5 vezes
16. Nos últimos 2 anos, já teve dificuldades com a aplicação dos remédios (não saber aplicar, não ter onde aplicar, dificuldade em usar as injeções, ou outras)?
    a. Não, nunca
    b. Sim, 1 vez
    c. Sim, 2 vezes
    d. Sim, de 3 a 5 vezes
    e. Sim, mais de 5 vezes

**Questões de atendimento médico:**

17. Nos últimos 2 anos, você teve alguma dificuldade em conseguir transporte para a consulta médica e, por isso, deixou de ir na consulta?
    a. Não, nunca
    b. Sim, 1 vez
    c. Sim, 2 vezes
    d. Sim, de 3 a 5 vezes
    e. Sim, mais de 5 vezes
18. Você mora na mesma cidade em que acompanha com o reumatologista?
    a. Sim
    b. Não, e preciso me deslocar 30-60 minutos
    c. Não, e preciso me deslocar de 1 a 2 horas
    d. Não, e preciso me deslocar 2 a 3 horas
    e. Não, e preciso me deslocar mais do que 3 horas
19. Com que frequência você consulta com o reumatologista?
    a. A cada 2-3 meses
    b. A cada 4 meses
    c. A cada 5-6 meses
    d. A cada 6-12 meses
    e. A cada 12-18 meses
20. Qual o intervalo entre as consultas que você consideraria ideal?
    a. A cada 2 meses
    b. A cada 3 meses
    c. A cada 4 meses
    d. A cada 5-6 meses
    e. A cada 6-12 meses
21. Nos últimos 2 anos, você saiu com dúvidas da consulta ou com assuntos que queria conversar, mas que não chegou a falar por falta de tempo?
    a. Não, nunca
    b. Sim, uma vez
    c. Sim, duas vezes
    d. Sim, de 3 a 5 vezes
    e. Sim, mais de 5 vezes
22. Nos últimos 2 anos, você apresentou efeito adverso de algum dos remédios (considerar cada efeito adverso de forma separada, mesmo que tenha sido vários efeitos do mesmo remédio)?
    a. Não, nunca
    b. Sim, uma vez
    c. Sim, duas vezes
    d. Sim, de 3 a 5 vezes
    e. Sim, e ainda apresento

**Questões de desinformação:**

23. Nos últimos 2 anos, houve algum sintoma que você teve e ficou bastante tempo sem saber que era relacionado à artrite psoriásica?
    a. Não, nunca
    b. Sim, uma vez
    c. Sim, duas vezes
    d. Sim, de 3 a 5 vezes
    e. Sim, mais de 5 vezes
24. Você sabia que não pode ingerir (beber) bebidas alcoólicas enquanto usa o metotrexato?
    a. Não, não sabia
    b. Sim, sabia
25. Você sabia que não pode engravidar enquanto usa o metotrexato (responde independente do sexo)?
    a. Não, não sabia
    b. Sim, sabia
26. Você sabia que é seguro engravidar e ter filhos mesmo tendo artrite psoriásica, desde que cuidando o momento ideal e as medicações em uso (responder independente do sexo)?
    a. Não, não sabia
    b. Sim, sabia

# **Table 3S.** Translated Questionnaire in English with Percentage of Answers per Question.

**Questions regarding diagnostic issues:**

1. How many months did it take for you to receive a psoriatic arthritis diagnosis after the onset of joint pain?

a. Less than 6 months – 11 (21.6%)

b. Between 6 and 12 months – 10 (19.6%)

c. Between 12 and 18 months – 7 (13.7%)

d. Between 18 and 24 months – 6 (11.8%)

e. More than 24 months – 17 (33.3%)

2. How many months did it take for you to get the first referral to a rheumatologist after the onset of joint pain?

a. Less than 6 months – 15 (29.4%)

b. Between 6 and 12 months – 10 (19.6%)

c. Between 12 and 18 months – 9 (17.6%)

d. Between 18 and 24 months – 3 (5.9%)

e. More than 24 months – 14 (27.5%)

3. How many months did it take for you to get an appointment with the rheumatologist after being referred by the family physician?

a. Less than 6 months – 20 (39.2%)

b. Between 6 and 12 months – 12 (23.5%)

c. Between 12 and 18 months – 9 (17.6%)

d. Between 18 and 24 months – 3 (5.9%)

e. More than 24 months – 7 (13.7%)

4. How many doctors did you see before receiving a diagnosis of psoriatic arthritis after the onset of joint pain?

a. Only 1 – 10 (19.6%)

b. 2 doctors – 18 (35.3%)

c. 3 doctors – 8 (15.7%)

d. 4 doctors – 4 (7.8%)

e. 5 or more doctors – 11 (21.6%)

**Questions related to difficulties in daily life:**

1. In the last 2 years, did joint pain or skin symptoms ever prevent or hinder you from performing day-to-day activities (cooking, cleaning the house ...)?

a. No, never – 3 (5.9%)

b. Yes, in the past – 11 (21.6%)

c. Yes, and they still hinder some activities – 15 (29.4%)

d. Yes, and they still hinder many activities – 11 (21.6%)

e. Yes, and they still hinder almost all of my activities – 11 (21.6%)

2. In the last 2 years, did joint pain or skin symptoms ever prevent or hinder you from performing physical activities (walking, gym, pilates)?

a. No, never – 4 (7.8%)

b. Yes, in the past – 7 (13.7%)

c. Yes, and they still hinder some activities – 24 (47.1%)

d. Yes, and they hinder many activities – 8 (15.7%)

e. Yes, and they hinder almost all physical activities – 8 (15.7%)

3. In the last 2 years, did joint pain or skin symptoms ever got in the way of your work?

a. No, never – 8 (15.7%)

b. Yes, in the past – 11 (21.6%)

c. Yes, and they still hinder some activities – 15 (29.4%)

d. Yes, and they hinder many activities – 8 (15.7%)

e. Yes, and they hinder almost all of my activities – 9 (17.6%)

4. In the last 2 years, did joint pain or skin symptoms ever made you request sick leave from work?

a. No, never – 14 (27.5%)

b. Yes, once – 4 (7.8%)

c. Yes, 2 to 5 times – 8 (15.7%)

d. Yes, more than 5 times – 7 (13.7%)

e. Yes, I retired because of the psoriatic arthritis (disability) – 18 (35.3%)

**Questions related to drug dispensation issues:**

1. How long did it take for you to receive the last medication you requested from the state pharmacy?

a. 1 or 2 weeks – 12 (23.5%)

b. 3 weeks – 6 (11.7%)

c. 4 weeks – 11 (21.6%)

d. 5 to 7 weeks – 10 (19.6%)

e. 8 weeks or more – 12 (23.5%)

2. In the last 2 years, have you had any difficulty in requesting medicines from the state pharmacy (lack of documents, specific papers or of recent exam, scheduling difficulties, etc.)?

a. No, never – 25 (49%)

b. Yes, once – 10 (19.6%)

c. Yes, twice – 8 (15.7%)

d. Yes, 3 to 5 times – 7 (13.7%)

e. Yes, more than 5 times – 1 (2%)

3. In the last 2 years, was your medication ever out of stock at the state pharmacy?

a. No, never – 21 (41.2%)

b. Yes, once – 6 (11.8%)

c. Yes, twice – 9 (17.6%)

d. Yes, 3 to 5 times – 10 (19.6%)

e. Yes, more than 5 times – 5 (9.8%)

4. In the last 2 years, have you ever been without medication because it was out of stock at the state pharmacy?

a. No, never – 23 (45.1%)

b. Yes, once – 10 (19.6%)

c. Yes, twice – 9 (17.6%)

d. Yes, 3 to 5 times – 6 (11.8%)

e. Yes, more than 5 times – 3 (5.9%)

5. In the last 2 years, did you have to legally request any medication (with a lawyer via legal process) because it was not available at the state pharmacy?

a. No, never – 39 (76.5%)

b. Yes, once – 9 (17.6%)

c. Yes, twice – 3 (5.9%)

d. Yes, 3 to 5 times – 0

e. Yes, more than 5 times – 0

6. If yes to the previous question (need for a legal process for medication access), how long did it take to get the medicine through legal means?

a. Never needed – 39 (76.5%)

b. 2 to 4 weeks – 2 (3.9%)

c. 4 to 8 weeks – 3 (5.9%)

d. 8 to 12 weeks – 2 (3.9%)

e. More than 12 weeks – 5 (9.8%)

7. In the last 2 years, have you had difficulties with the transportation of medicines (not knowing the necessary care needed, not having styrofoam, the pharmacy being too far, etc.)?

a. No, never – 46 (90.2%)

b. Yes, once – 3 (5.9%)

c. Yes, twice – 2 (3.9%)

d. Yes, 3 to 5 times – 0

e. Yes, more than 5 times – 0

8. In the last 2 years, have you had difficulties with the application of medicines (not knowing how to apply, not having a place to go to apply, difficulty in using injections, etc.)?

a. No, never – 46 (82.3%)

b. Yes, once – 6 (11.8%)

c. Yes, twice – 1 (2%)

d. Yes, 3 to 5 times – 0

e. Yes, more than 5 times – 2 (3.9%)

**Questions related to issues with routine medical care:**

1. In the last 2 years, have you had difficulty getting transportation for the medical appointment and, as a result, missed the appointment?

a. No, never – 42 (82.3%)

b. Yes, once – 6 (11.8%)

c. Yes, twice – 3 (5.9%)

d. Yes, 3 to 5 times – 0

e. Yes, more than 5 times – 0

2. Do you live in the same city where you see your rheumatologist?

a. Yes – 17 (33.3%)

b. No, and I need to travel 30-60 minutes – 9 (17.6%)

c. No, and I need to travel 1 to 2 hours – 9 (17.6%)

d. No, and I need to travel 2 to 3 hours – 7 (13.7%)

e. No, and I need to travel more than 3 hours – 9 (17.6%)

3. How often do you see your rheumatologist?

a. Every 2-3 months – 18 (35.3%)

b. Every 4 months – 10 (19.6%)

c. Every 5-6 months – 17 (33.3%)

d. Every 6-12 months – 6 (11.8%)

e. Every 12-18 months – 0

4. What interval between visits do you consider ideal?

a. Every 2 months – 6 (11.7%)

b. Every 3 months – 15 (29.4%)

c. Every 4 months – 11 (21.6%)

d. Every 5-6 months – 17 (33.3%)

e. Every 6-12 months – 2 (3.9%)

5. In the last 2 years, did you leave the appointment with doubts or issues you wanted to discuss but didn't get the chance due to time constraints?

a. No, never – 42 (82.3%)

b. Yes, once – 5 (9.8%)

c. Yes, twice – 1 (2%)

d. Yes, 3 to 5 times – 3 (5.9%)

e. Yes, more than 5 times – 0

6. In the last 2 years, did you experience side effects from any of the medications prescribed for psoriatic arthritis (consider each side effect separately, even if there were multiple effects from the same medication)?

a. No, never – 26 (50.9%)

b. Yes, once – 10 (19.6%)

c. Yes, twice – 3 (5.9%)

d. Yes, 3 to 5 times = 6 (11.8%)

e. Yes, and I still experience them (6 (11.8%)

**Questions related to misinformation:**

1. In the last 2 years, was there any symptom you experienced and took a long time to realize it was related to psoriatic arthritis?

a. No, never – 28 (54.9%)

b. Yes, once – 13 (25.5%)

c. Yes, twice – 2 (3.9%)

d. Yes, 3 to 5 times – 5 (9.8%)

e. Yes, more than 5 times – 3 (5.9%)

2. Were you aware that you shouldn't consume (drink) alcoholic beverages while taking methotrexate?

a. No, I wasn't aware – 27 (27.5%)

b. Yes, I was aware – 37 (72.5%)

3. Were you aware that you shouldn't get pregnant while taking methotrexate (answer regardless of gender)?

a. No, I wasn't aware – 19 (37.3%)

b. Yes, I was aware – 32 (62.7%)

4. Were you aware that it is safe to get pregnant and have children even with psoriatic arthritis, as long as you're mindful of the ideal timing and the medications being used (answer regardless of gender)?

a. No, I wasn't aware – 20 (39.2%)

b. Yes, I was aware – 31 (60.8%)
